# Dynamics of recruitment and establishment of the invasive seaweed *Codium fragile* within an eelgrass habitat

**DOI:** 10.1007/s00227-016-2832-z

**Published:** 2016-02-24

**Authors:** Annick Drouin, Christopher W. McKindsey, Ladd E. Johnson

**Affiliations:** Demersal and Benthic Sciences Branch, Maurice-Lamontagne Institute, Fisheries and Oceans Canada, PO Box 1000, Mont Joli, QC G5H 3Z4 Canada; Département de biologie et Québec-Océan, Université Laval, Quebec, QC G1V 0A6 Canada

## Abstract

Knowledge of the potential distribution (i.e. abundance and spatial extent) of an invasive species is important to estimating its potential impacts on recipient communities. Most previous studies have focused on the potential spatial extent of invasive species populations at regional scales, but little is known on how species successfully recruit and establish at more local scales. In this study, we examined how recruitment of the green alga *Codium fragile* ssp. *fragile* (hereafter *Codium*) can vary spatially and the environmental factors associated with *Codium* establishment in eelgrass (*Zostera marina*) beds. Standardized recruitment blocks (65 blocks in a 720 × 240 m^2^ grid) were used to monitor the number of *Codium* recruits, juveniles and adults over 2 years. Environmental factors (depth, relative water flow, light and temperature) and attributes of the surrounding macrophyte assemblage (eelgrass density, eelgrass length, *Codium* biomass) were also measured. Recruitment occurred on all blocks or nearby artificial structures (i.e. buoys) and mainly originated from button stages (i.e. female gametes or utricles). Contrary to other studies, the abundance of *Codium* (recruits, juveniles and adults) was best predicted by the density of the native canopy-forming species, *Z. marina*, which highlights a positive interaction between native and non-native canopy-forming species. Seasonal variation in recruitment was observed; it was lower during the summer. Recruitment did not show any distinct spatial pattern (e.g. gradient or patch), but the same spatial pattern of recruitment was observed every sampling date, suggesting that there are “hotspots” for recruitment. In general, the total number of *Codium* fronds observed on a block at the end of the experiment was positively correlated with the cumulative number of recruits. However, recruitment occurred on some blocks but recruits never grew, suggesting that some environmental factors limit *Codium* distribution and abundance in eelgrass beds. Overall, the assessment of *Codium* recruitment over 2 years showed that the colonization of suitable locations by *Codium* within seagrass beds may take several years and that some factors may not only limit, but also inhibit *Codium* expansion within eelgrass beds.

## Introduction

Much effort has focused on identifying factors limiting invasion success over global and regional scales, with the goal of predicting the rate of range expansion and identifying vectors of dispersion (e.g. Lyons and Scheibling 2009; Johnson et al. 2012; Jofré Madariaga et al. 2014; Gagnon et al. 2015b). In contrast, little information is available on how invasive species successfully disperse and establish at local scales (but see Sepulveda and Marczak 2012). Patterns in invasibility may differ greatly from landscape- to small-scales, as well as temporaly, largely because mechanisms that influence species distributions are not homogenous through space and time (e.g. Davis et al. 2000; Pyšek and Hulme 2005). Invasion dynamics have similar steps at local, regional and global scales, although the mechanisms may differ greatly. Species first need to reach a new area (arrival/recruitment) and then pass through various abiotic and biotic filters (establishment/colonization) so that a local population may thereafter expand within and to new locations (spread/distribution). The local extent of an invasive species population is thus dependent on coupled dispersal–recruitment dynamics and subsequent interactions with the recipient community and the local environment.

Space monopolization of native habitat is one of the main ecological effects reported for invasive seaweeds (Davidson et al. 2015). However, this does not necessarily result in a “hostile takeover”. In îles de la Madeleine, eastern Canada, the invasive green seaweed *Codium fragile* ssp. *fragile* (synonymous of ssp. *tomentosoides*, Provan et al. 2008, and hereafter *Codium*) is observed to grow epiphytically on eelgrass (*Zostera marina*) rhizomes. Hydrodynamic processes may contribute to expose rhizomes and encourage the horizontal growth as a phenotypic response by *Codium *to growing in soft-bottom habitats (Garbary et al. 2004) and may explain the association of eelgrass with *Codium.* At the time of our study, *Codium* had already formed extensive stands within some eelgrass beds in the study site and had likely been present for many years prior to its initial detection in 2003 (Simard et al. 2007). However, *Codium* did not occur at a uniformly high biomass throughout the invaded area (A. Drouin, pers. obs.). Given that field experiments have shown that a high density of *Codium* has negative effects on eelgrass (Drouin et al. 2012) and influences associated invertebrate and fish communities (Drouin et al. 2011), knowledge of the mechanisms that influence the occurrence and spatial extent of *Codium* abundance within eelgrass habitat will allow a better assessment and understanding of its ecological impacts (Kolar and Lodge 2001; Strayer et al. 2006; Olenin et al. 2007; Thiele et al. 2010).

The dispersal capacity of invaders is of prime interest in the study of recruitment patterns as propagules must first reach an area to settle. Spread of *Codium* may occur by the dispersal of parthenogenic female gametes (Trowbridge 1998; Prince and Trowbridge 2004), isolated utricles (the loosely compacted filaments that make up the thallus), or vegetative buds or thallus fragments (Fralick and Mathieson 1972; Trowbridge 1998; Nanba et al. 2002). While dislodgment of buds, fragments, or thalli may contribute to *Codium* dispersion over several kilometres (Gagnon et al. 2011, 2015a), female gametes have lower mobility, swimming for only a few minutes after they have been released (Coolidge Churchill and Moeller 1972), and thus, settlement should be expected to occur predominantly close (within metres) to adults. As with rafting seaweeds (e.g. *Durvillaea antarctica*, Thiel and Gutow 2005), drifting *Codium* may act as a source of propagules (e.g. female gametes or utricles) and allow for dispersal to occur over greater distances than it would from thalli attached to the substrate. Unattached plants may also persist within the low-energy hydrodynamic environments, such as eelgrass habitats, and continue to contribute to demographic processes (Gagnon et al. 2011, 2015a). The range over which *Codium* might spread is dependent on its reproductive mode and dispersal rate, which may be influenced by environmental conditions (D’Amours and Scheibling 2007). Thus, spatial structure in *Codium* recruitment should provide insight into the importance of different dispersal modes for the alga, but also reflect niches within an area (Shea and Chesson 2002). For example, a patchy pattern of recruitment within a population may result from spread due to a limited dispersal mode (gametes) such that individuals recruit near attached parent plants, or because environmental conditions that permit establishment and growth tend to be spatially auto-correlated.

Following the arrival of propagules, some environmental parameters (such as resource availability, interspecific relationships and physical conditions) may promote invasion success, whereas others may limit the establishment of non-native species at a local scale (Melbourne et al. 2007). In seagrass habitats, high sedimentation rates and low light levels or shading by leaves may limit *Codium* growth (Malinowski and Ramus 1973; Thomsen and McGlathery 2007; Thomsen et al. 2007) and thus negatively influence its distribution. Conversely, the availability of substrata is an important resource that can stimulate invasion by this species. Gagnon et al. (2015a) observed that disturbance can promote the establishment of *Codium* in eelgrass beds, likely because it increases the availability of suitable substrata by exposing rhizomes. The same study also noted that eelgrass density may promote invasion by increasing the retention of drifting fragments.

Temporal variation in effects may also be expected when studying invasion success. Spread within a given location is logically a function of the time since initial establishment, although it may not occur at a regular rate or follow a simple diffusive pattern. Moreover, invasive species success can fluctuate through time (Simberloff and Gibbons 2004). As observed by Drouin et al. (2012), environmental factors can influence the persistence of *Codium* in a given area and thus its pattern of distribution through time.

The aim of this study was to evaluate the importance of spatial and environmental factors on the recruitment and establishment of *Codium* within an invaded eelgrass bed to better understand its distribution at small spatial scales. Specifically, we assessed spatial variation in *Codium* recruitment in eelgrass beds and the environmental factors that are correlated with *Codium* establishment there. To this end, we examined the relationship between environmental conditions and the distribution of *Codium* recruitment and establishment on regularly distributed standardized substrata over 2 years in an invaded eelgrass meadow.

## Methods

### Study site

**Fig. 1:**
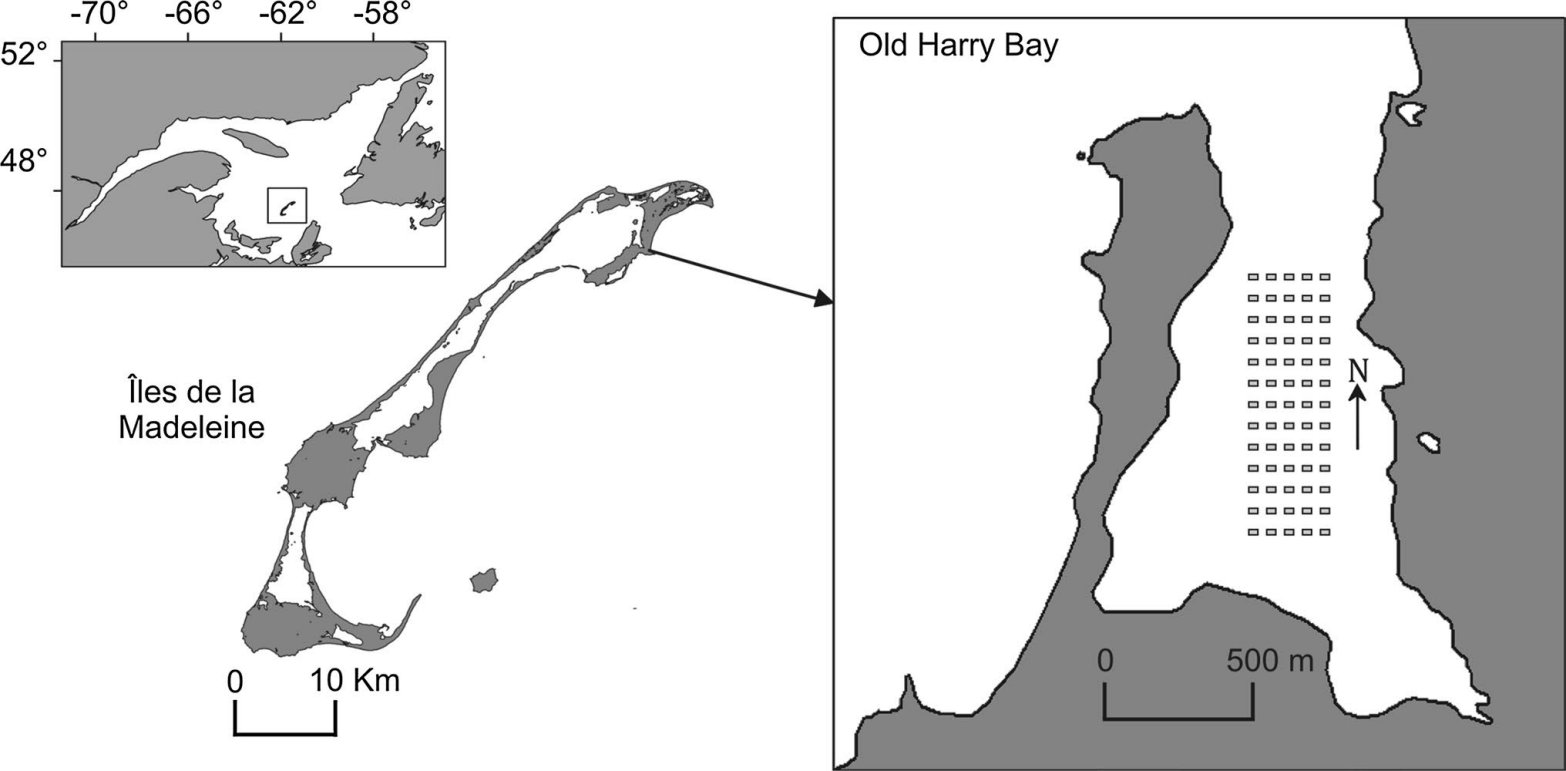
Location of the study area, îles de la Madeleine, Eastern Canada, and spatial arrangement of the 65 recruitment blocks (*grey rectangles*) spaced at 60-m intervals in Old Harry Bay

This study was conducted in Old Harry Bay, an embayment of the Grande-Entrée Lagoon, îles de la Madeleine, Eastern Canada (Fig. 1). The region is characterized by low amplitude tides (0.60 m) with salinity stable at around 30 ‰ as precipitation is the only source of fresh water. Calm conditions are infrequent, and the currents and waves generated by local winds are the main factors mixing the water column in shallow areas (Koutitonsky et al. 2002). Water temperature can exceed 20 °C in summer and is below 0 °C during the winter (December to April). *Codium* was first observed in this area in 2003 growing on eelgrass rhizomes, although the extent of the population at that time indicated that it was already well established (Simard et al. 2007) and had likely been present for at least 3–5 years before this first observation. At the time of our study, the distribution of *Codium* within eelgrass beds was a mosaic with densities varying at different spatial scales such that more or less pure eelgrass areas were interspersed with areas up to ca. 25 m^2^ that were largely dominated by *Codium* (Drouin et al. 2011).

### Recruitment pattern

Although *Codium* most typically grows on eelgrass rhizomes at this site, we used hollow concrete blocks (0.40 m long, 0.20 m wide and 0.15 m high—henceforth “blocks”) as a standardized substratum to monitor *Codium* recruitment. Each block was marked by a 5-cm buoy that was positioned ca. 20 cm above the eelgrass canopy and anchored with a 20-cm nail driven into the seafloor ca. 20 cm away from each block to facilitate finding them for sampling. Elsewhere, *Codium* grows primarily on hard substrata (see above), and it had earlier been observed growing on similar concrete blocks at this site, so it was known that it could settle and grow on this type of surface. Recruitment was assessed over time and space by counting the number of recruits, i.e. settlers that had reached a “button stage” (Sears and Wilce 1970; Ramus 1972)—an erect macroscopic thalli with a single primordial axis of about 0.5–1 cm (Fig. 3). This stage follows the initial undifferentiated filamentous phase of *Codium* development (Ramus 1972) and was easily distinguishable with the naked eye in the field; moreover, this distinction avoided potential confusion with other filamentous green algae. According to Nanba et al. (2002), a single utricle can potentially become a new individual, reaching button size in 10 weeks, but these recruits could also have resulted from settlement of parthenogenic gametes.

**Fig. 2:**
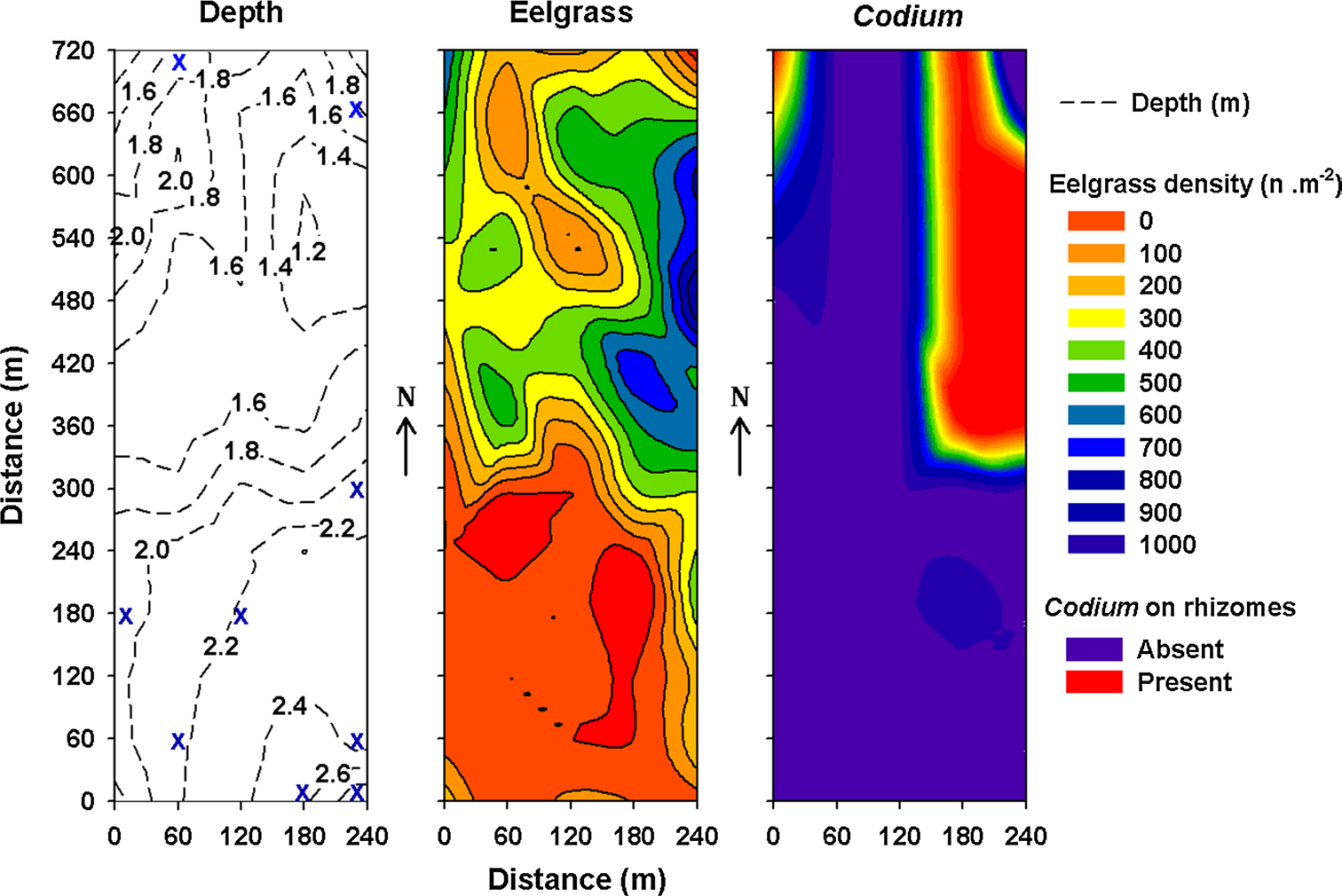
Gradient of depth (m), eelgrass density (n m^−2^) and location of *Codium* growing on eelgrass rhizomes within the grid area, oriented north–south, in July 2007. The *blue* cross within the depth panel (*n* = 9) represents the blocks where some recruitment occurred, but no establishment of *Codium* was observed at the end of the study

**Fig. 3:**
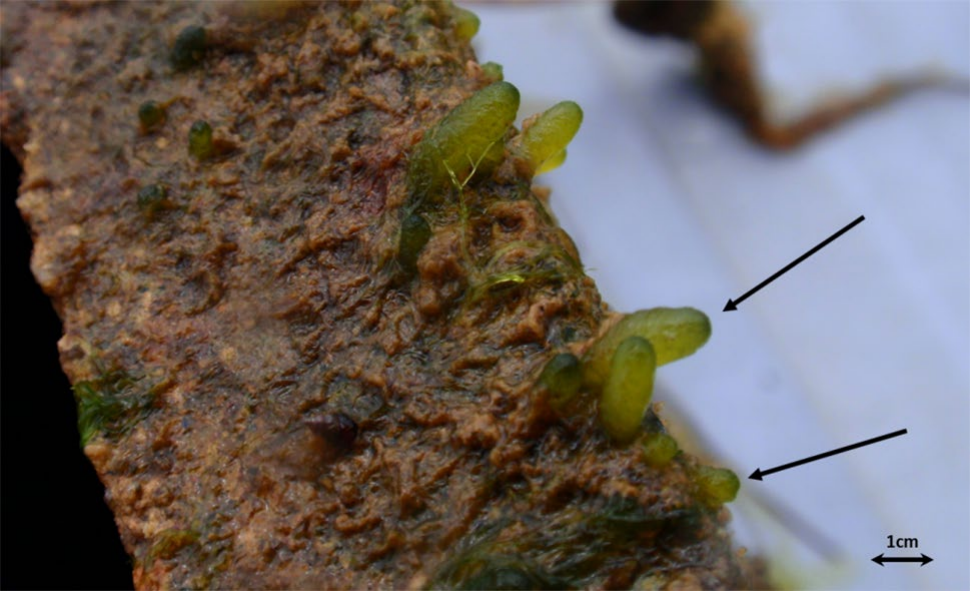
Button stages of *Codium* growing at the edge of a recruitment block

In July 2007, 65 blocks were placed at 60-m intervals within a regular 720 × 240 m^2^ grid (Fig. 1). The grid was positioned to include eelgrass beds varying in their degree of invasion, ranging from areas where *Codium* grew attached to eelgrass rhizomes in more or less defined patches to non-invaded eelgrass beds, where only occasional drifting fragments of *Codium* were observed (Fig. 2). Each block was later sampled using scuba diving at four times (October 2007; June 2008; September 2008; and June 2009). *Codium* recruits and larger fronds on blocks were counted on each sampling date from October 2007. Since reproduction is observed on thalli from 10 to 12 cm (Coolidge Churchill and Moeller 1972), fragments less than 1 cm in length were considered recruits (Fig. 3), those >1–10 cm juveniles and those 11–30 cm adults. The abundances of juveniles and adults were used as proxies to assess the survival and growth of the recruits and juveniles, respectively, between sampling periods. At the end of the experiment, 20 thalli from each length class were collected haphazardly from the blocks and weighed after excess water was removed using a salad spinner. The biomass of *Codium* on the blocks at each sampling period was then estimated from the regression of *Codium* wet weight as a function of length. It was impossible to recover some of the blocks (one in October 2007, three in June 2008, four in September 2008 and 15 in June 2009); the exact cause of their loss remains unknown.

### Environmental conditions

Water depth was measured at each recruitment block (time and date were noted for each measurement to later standardize the mean water depth according to the tide level using the tide table for the study area). Relative water flow was estimated by measuring the mass loss of plaster of Paris cylinders (9 cm long, 3 cm diameter; Lepage™, Brampton, Ontario, Canada) placed over a metal rod attached vertically to the corner of each recruitment block. The ends of the cylinders were covered with a layer of fibreglass resin to prevent dissolution at cylinder ends to ensure that a near-constant area was exposed during deployment. The plaster cylinders were deployed for 24 h (two full semi-diurnal tidal cycles) and dry mass loss (initial–end weight) used as a relative index of hydrodynamic action (rate of plaster loss, flow index, g d^−1^; Thompson and Glenn 1994). Plaster cylinders were deployed on three haphazardly selected dates in July 2007. For two of the dates, winds reached speeds higher than 50 km h^−1^, which is representative of the conditions that may occur in the studied area during the summer. Variation in light and temperature among recruitment blocks was evaluated using 12 Onset HOBO pendant light and temperature loggers (model UA-002-64) that were deployed from 17 June to 6 September 2008. Loggers were installed on stakes at the surface of the blocks to measure light coming from the surface and were distributed among stations to cover the entire range of depths found in the study area (i.e. from 1.2 to 3.0 m). Light data were used to estimate the period of day when light intensity exceeded the light saturation and compensation levels for *Codium* (Arnold and Murray 1980).

### Macrophyte attributes

The vegetation in the vicinity of each recruitment block was characterized in July 2007 and July 2008. Eelgrass shoot density was estimated within 0.1-m-diameter-circular plots (0.008 m^−2^) randomly placed within 5 m of each block (*n* = 8 per block). Maximum eelgrass length was measured haphazardly at three places in these same areas using a measuring tape. *Codium* biomass was measured by collecting all the fronds within three 0.5 × 0.5-m^2^ haphazardly placed quadrats within a zone from 1 to 5 m around each block. *Codium* thalli were promptly weighed after removing excess water using a salad spinner. A major unanticipated shift in the distribution of *Codium* occurred during the study. Great quantities of large thalli previously attached to rhizomes were observed scattered all over the study site in June 2008, and thus, the status of *Codium* was also noted, i.e. attached to eelgrass rhizomes or drifting specimens, and used as a binary variable for subsequent analyses.

### Statistical methods

Distance–recruitment relationships for recruitment blocks were determined with a measure of spatial autocorrelation, Moran’s *I,* plotted against distance (m, Fortin and Dale 2005). Correlograms based on Moran’s *I* were created for *Codium* recruit, juvenile and adult abundance on recruitment blocks using SAM 3.0 (Rangel et al. 2010). The number of distance classes was determined using Sturge’s rule based on sample size and an equal-number-of-pairs approach to minimize edge effects (Fortin and Dale 2005). The test of significance of the entire correlogram was determined using the Bonferroni correction (Fortin and Dale 2005). Kendall’s coefficient (*τ*) was used to test for the presence of monotonic relationships (i.e. a variable maintaining the same given order over time) for the same independent variables between two sampling dates, and for measures of the association between the abundance of a specific class size at a specific sampling date and the subsequent class size at the following sampling date (Quinn and Keough 2002).

To extrapolate the light data recorded at 12 blocks to the entire grid of blocks, the relationships between light intensity, depth, water flow and eelgrass attributes were evaluated using regression models. The relationships between the cumulative number of recruits observed on blocks before June 2009 and the total number of growing fronds (>10 cm) at the end of the experiment, i.e. in June 2009, were also evaluated using regression models. The relationships between environmental data and *Codium* recruit, juvenile and adult abundances was tested for each sampling date using distance-based linear models (DISTLM) in PERMANOVA+ for PRIMER-e version 6 (Anderson et al. 2008). Conditional tests using forward selection were used to evaluate the proportion of variability in the abundance of *Codium* recruits or growing fronds explained by the environmental data while controlling for covariance among independent variables. Analyses were performed on raw data because initial transformations did not improve the fit of data to assumptions and DISTLM is robust to departure from statistical assumptions because of the permutational approach employed. Environmental variables used in the models were depth, flow index, eelgrass shoot density and length, biomass of *Codium* in the vicinity of the blocks, presence/absence of *Codium* attached to eelgrass rhizomes surrounding the blocks, and total biomass of *Codium* on the blocks at the preceding sampling periods. Collinearity among predictors was evaluated by examining tolerance values (i.e. above a reference point of 0.1) (Quinn and Keough 2002) but was absent except between eelgrass density in 2007 and 2008 and eelgrass length in 2007 and 2008, so the 2007 values were removed from subsequent analyses. Spearman coefficient (rho) (or in the case of presence/absence of *Codium* attached to eelgrass rhizomes, the point biserial coefficient, *r*_pb_) was used to test the correlation among the best predictor variables obtained from DISTLM results and dependent variables. ANOVAs were used to compare environmental characteristics (eelgrass density, eelgrass length, flow index and depth) among groups of recruitment blocks following evaluating the assumption of homoscedasticity using Bartlett’s test. *A posteriori* comparisons were made using Tukey’s tests.

## Results

### Pattern of *Codium* establishment

**Fig. 4:**
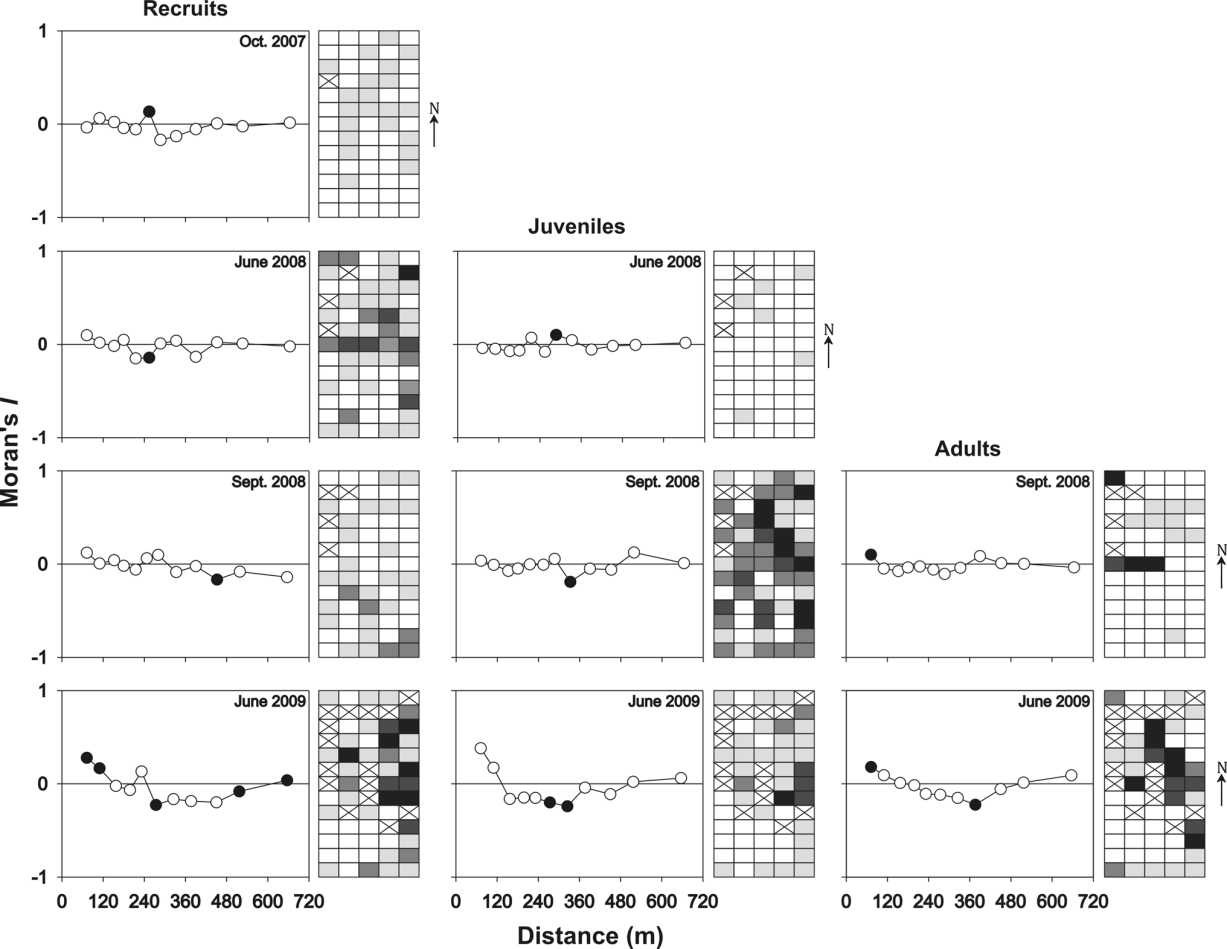
Spatial structure of the abundance *Codium* recruits, juveniles and adults at each sampling date. Schemas (*right*) are oriented north–south and show a simplified mapping of *Codium*’s abundance on recruitment blocks. The *intensity of colours* denotes the abundance of *Codium* (0; 1–25; 26–50; 51–75; 76–100+, from *white* to *black*, respectively) on blocks, and *crosses* indicate missing blocks. Graphs (*left*) are spatial correlograms showing Moran’s *I* by distance (m). *Black circles* indicate significant Moran’s *I* values (*p* < 0.05, based on 999 permutations)

Few drift fragments were observed to have recruited onto blocks and none of these were observed at the following sampling date. Thus, the observed growing fronds mainly originated from button stages (female gametes or utricles) and almost all the recruitment occurred along upper block margins. The proportion of blocks with *Codium* growing on them was greatest in September 2008, 14 months after the blocks were first set out, followed by a decrease at the beginning of the next growing season (Table 1). At the end of the experiment (i.e. June 2009), *Codium* was absent from the surface of 13 blocks (26 %), but recruits were observed on all the submerged buoys used to locate the blocks. The abundance of recruits observed on the blocks was greater in June 2008 and 2009 than in September 2008; the abundance of juvenile *Codium* decreased between September 2008 and June 2009, while the abundance of adults increased from September 2008 to June 2009 (Fig. 4). Temporally, the abundance of recruits on blocks increased between October 2007 and June 2008, as well as between September 2008 and June 2009, but decreased between June 2008 and September 2008. The abundance of recruits, juveniles and adults varied spatially but showed no clear significant spatial pattern (i.e. patchiness or gradient, Fig. 4). There was a significant correlation in the abundance of recruits observed in October 2007 and that observed in June 2008 (*τ* = 0.27, *p* = 0.011), as well as between June 2008 and June 2009 (*τ* = 0.32, *p* = 0.004). Juvenile abundance in September 2008 was also correlated to that observed in June 2009 (*τ* = 0.29, *p* = 0.0051). Significant associations were also observed between the abundance of recruits observed in June 2008 and the abundance of juveniles and adults in September 2008, the following sampling date (Fig. 4; Table 2).

**Table 1:** Proportion of recruitment blocks (%) with *Codium* at each sampling date. *Numbers in brackets* represent the number of recruitment blocks assessed

Dates	Proportion
October 2007	36 % (64)
June 2008	65 % (62)
September 2008	83 % (61)
June 2009	72 % (50)

**Table 2:** Measure of the association (Kendall tau) between abundance of a class size of *Codium* and the next greater class size at the subsequent sampling date

Pairs of individual size and date		Kendall tau
*x*	*y*	
Recruits, October 2007	Juveniles, June 2008	0.16
Juveniles, June 2008	Adults, Sept. 2008	0.16
Recruits, June 2008	Juveniles, Sept. 2008	**0.39**
Juveniles, September 2008	Adults, June 2009	**0.26**
Recruits, September 2008	Juveniles, June 2009	0.16

Bold values indicate significant correlations

### Factors linked with *Codium* establishment

**Fig. 5:**
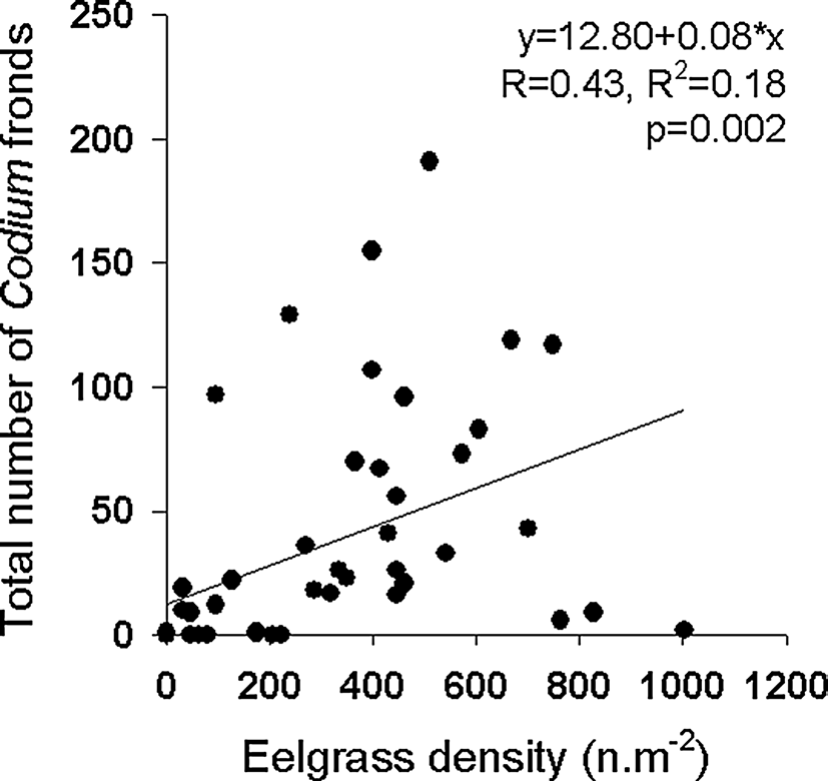
Relationship between the eelgrass density observed within a radius of 5 m from the recruitment blocks and the total number of fronds observed on the blocks in June 2009

**Fig. 6:**
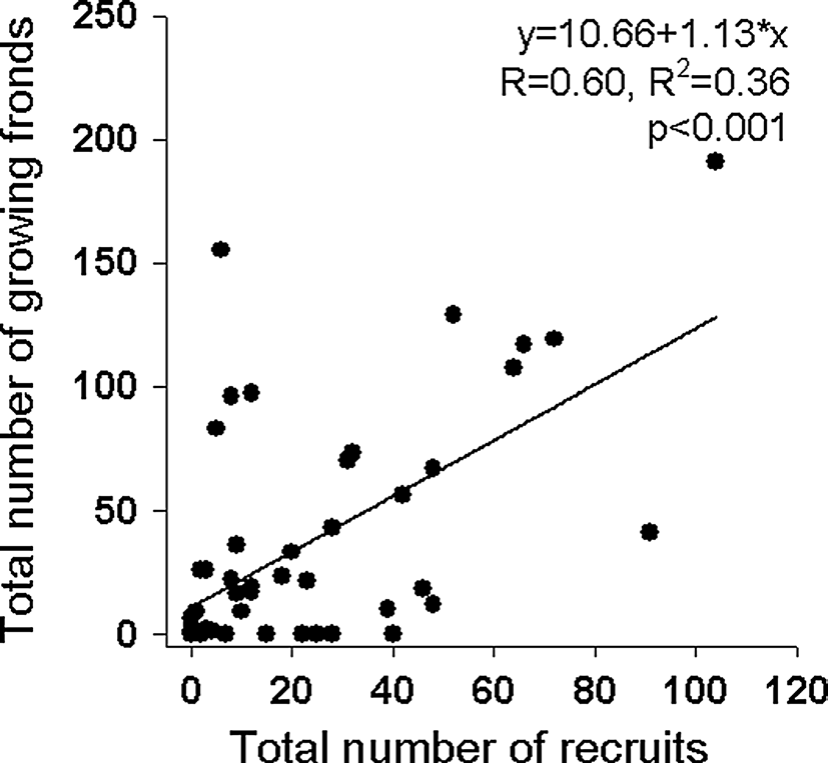
Relationship between the total number of recruits observed before June 2009 and the total number of growing fronds observed in June 2009 on the recruitment blocks

**Fig. 7:**
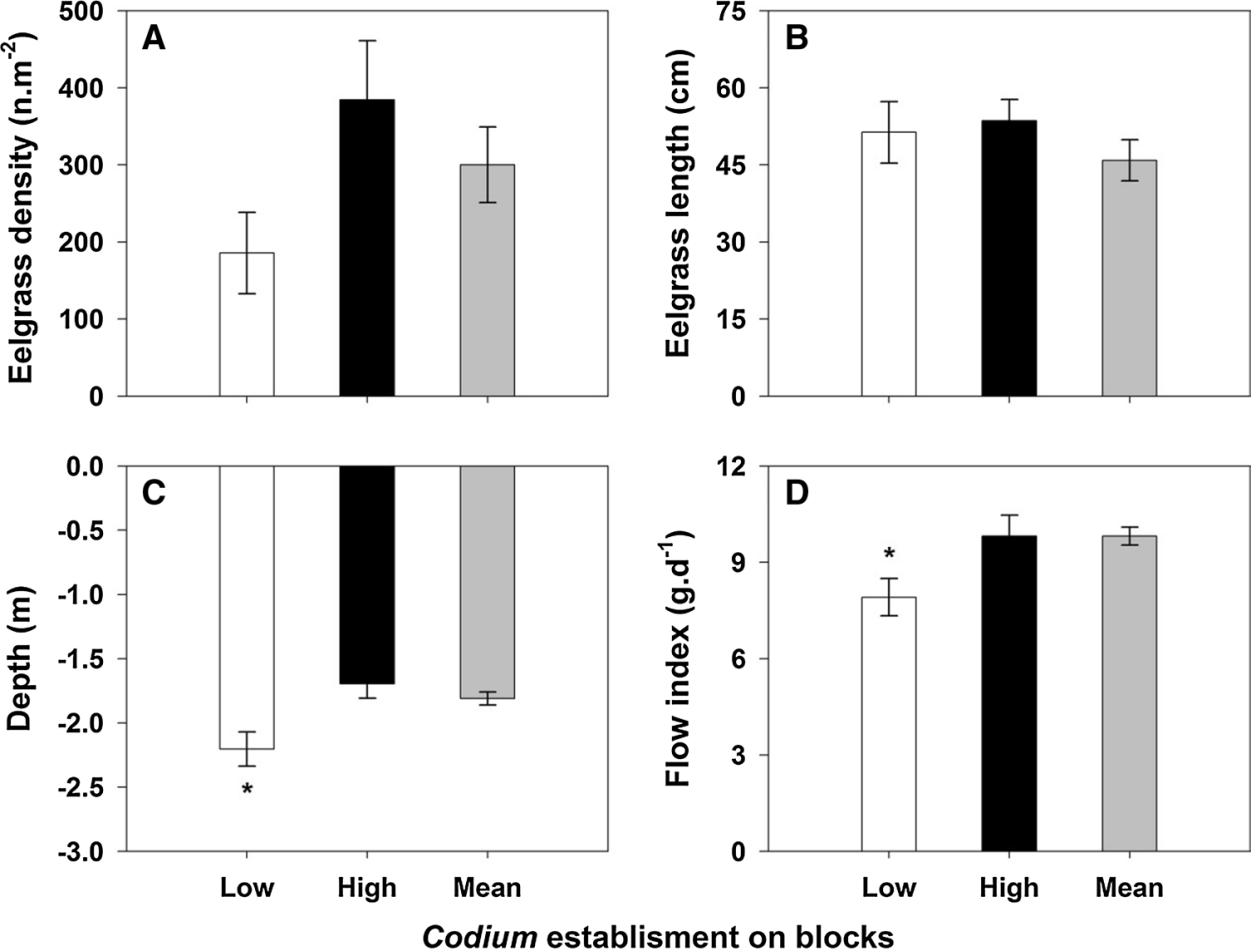
**a** Eelgrass density in 2009, **b** eelgrass length in 2009, **c** depth and **d** flow index (mean ± SE) from group of blocks in areas with low (*white*, *n* = 9), high (*black*, *n* = 6) or close to 1 (mean, *grey*, *n* = 50) *Codium* fronds: recruits ratios according to Fig. 5. *Asterisks* indicate significant differences between ratio treatments according to Tukey’s test (*p* < 0.05)

Temperature was similar at the 12 recorded stations, with a mean daily temperature of 19.7 ± 2.7 °C (min: 13 °C; max: 25 °C), and thus, this variable was not investigated further. The number of hours at saturation and daily compensation levels varied among the 12 recorded stations (daily mean: 5.5 ± 1.3 and 11.0 ± 0.9 h day^−1^, respectively), but was not significantly correlated with depth, water flow, or eelgrass density or length, thus preventing any extrapolation from these 12 blocks to the whole set of blocks for these variables. In addition, the correlations between the total *Codium* biomass on this subset of blocks at the end of the experiment and cumulative values of light periods were not significant (saturation level: *R* = 0.03, *p* = 0.920; compensation level: *R* = 0.01, *p* = 0.978; *n* = 12). Predictors of the abundance of the different stages of *Codium* varied among sampling dates (Table 3). Biomass of *Codium* on blocks at the previous sampling date and eelgrass density were both positively correlated with the number of *Codium* and accounted for the greatest proportion of the explained variation in models (Table 3). A positive correlation was observed between eelgrass density near the recruitment blocks and the total number of fronds observed growing on the blocks at the end of the monitoring in June 2009 (Fig. 5). The total number of recruits observed on blocks before June 2009 (i.e. cumulative counts for each block) and the total number of fronds (i.e. juveniles and adults) at the end of the experiment in June 2009 were positively correlated (Fig. 6). For the majority of blocks, the number of recruits was a good indicator of the number of mature fronds growing on them at the end of the experiment (*m* = 1, Fig. 6). However, on some blocks (*n* = 9) where recruitment was observed, recruits never reached an adult size, as no fronds were observed on the blocks at the end of the experiment, while at other blocks (*n* = 6), the number of growing fronds was 8–26 times higher than predicted by the number of recruits (Fig. 6). Comparisons of environmental characteristics around the blocks with “low” and “high” *Codium* establishment (Fig. 7) showed that the group of blocks with a lower establishment were associated with deeper areas (*F*_2,62_ = 0.008, *p* = 0.008) and lower flow indices (*F*_2,62_ = 3.432, *p* = 0.039). The mean eelgrass density at the end of the experiment (June 2009) was also lower in the vicinity of those blocks, but this trend was not statistically significant (*F*_2,62_ = 1.128, *p* = 0.332). The group of recruitment blocks with a high ratio of growing fronds to recruits had environmental characteristics that were similar to those of blocks with a ratio of fronds to recruits of ca. 1 (Fig. 7).

**Table 3:** Results of the DISTLM test on *Codium* abundance for each class sizes (recruits, juveniles and adults) at each sampling date and correlation coefficients (Spearman, rho, or point biserial, *r*_pb_) among predictor and dependant variables

Dates	Abundance	Best predictors	AIC	SS	Pseudo-*F*	*p* values	%Var	%Cum	rho/*r*_pb_
October 2007	Recruits	Flow	130.4	30.7	3.22	0.090	5.54	5.54	−0.09
		Depth	128.4	35.6	3.94	0.057	6.42	11.96	−0.15
June 2008	Recruits	Eelgrass density	310.1	4383.4	13.10	**0.001**	20.43	20.43	**0.58**
		Flow	307.9	1286.3	4.08	0.059	6.00	26.43	−0.15
	Juveniles	Flow	30.1	4.1	2.40	0.141	4.50	4.50	−0.11
		*Codium* biomass on blocks in Oct. 2007	29.8	3.7	2.21	0.060	4.05	8.55	**0.42**
September 2008	Recruits	*Codium* biomass around blocks in July 2008	41.3	0.7	0.36	0.496	0.66	0.66	0.03
	Juveniles	*Codium* biomass on blocks in June 2008	387.1	17134.0	17.66	**0.001**	24.64	24.64	**0.59**
		Eelgrass length	382.2	6047.7	6.92	**0.007**	8.70	33.34	**0.35**
		Presence of attached *Codium*	381.2	2467.1	2.92	0.117	3.55	36.89	0.21
	Adults	Depth	364.4	3458.3	5.34	**0.033**	9.01	9.01	−**0.52**
		*Codium* biomass on blocks in June 2008	363.0	2097.7	3.38	0.059	5.46	14.47	**0.30**
June 2009	Recruits	Eelgrass density	316.8	6934.2	6.35	**0.014**	12.87	12.87	**0.51**
		*Codium* biomass on blocks in Sept. 2008	315.9	2908.7	2.77	0.100	5.40	18.26	0.26
		*Codium* biomass around blocks July 2008	315.0	2779.9	2.76	0.102	5.16	23.42	0.08
	Juveniles	Eelgrass density	267.1	2189.7	6.04	**0.013**	12.32	12.32	**0.61**
	Adults	*Codium* biomass on blocks in Sept. 2008	285.4	47701.0	87.64	**0.001**	67.09	67.09	**0.78**

Bold values indicate significant correlations

*AIC* Akaike information criterion, *SS* sum of squares, *%Var* percentage of variance explained by a given variable, *%Cum* cumulative per*c*entage of variance explained

## Discussion

The results of the present study showed that *Codium* dispersal occurred across the entire study area, as recruits were observed on all blocks or nearby artificial structures (i.e. buoys) at some point during the study period. However, *Codium* abundance varied over space and time and was best predicted by the density of the native canopy-forming species, *Z. marina*. This positive correlation contrasts with previous observations of seaweed invasion in seagrass beds (Ceccherelli and Cinelli 1997; den Hartog 1997; Ceccherelli et al. 2000) and *Codium* invasions of rocky shores (Scheibling and Gagnon 2006; Valentine et al. 2007), where gaps and low macrophyte density promoted the invasion. The observations from the present study highlight that invasibility may be sustained by facilitation interactions (Fridley et al. 2007; Bulleri et al. 2008). The assessment of recruitment over 2 years shows that the colonization of suitable locations by *Codium* within seagrass beds may take several years and that some factors may not only limit, but also inhibit *Codium* expansion within eelgrass beds.

### Dispersal and recruits pattern

Despite the presence of drift fragments and thalli within the studied bay, *Codium* growing on blocks was mostly at the button stage, and thus* Codium* colonization was likely primarly due to gametes or utricles during this study. Drifting fragments might also have indirectly contributed to dispersal by transporting gametes and utricles, the later which may be released from damaged fronds or through abrasion of the frond on blocks. Interestingly, recruits were mainly observed around the edges of the blocks, which have resulted via several mechanisms, such abrasion of fronds, differential settlement of propagules (Johnson 1994), or by hydrodynamic effects created by the blocks. In the same area, Gagnon et al. (2015a) observed low variation among *Codium* thallus length 10 months after recruitment, indicating that individuals were likely from a same cohort and thus had not originated from the settlement of drifted fragments. Given the lack of correlation between the density of recruits and the local abundance of *Codium*, the hypothesis that drift fragments contribute to the transport of gametes and utricles is possible, or that gametes may have a higher dispersal range than previously reported (e.g. Trowbridge 1998; Prince and Trowbridge 2004). Since the abundance of drifting *Codium* varies with patterns of water movement and the seasonal production of fragments (Gagnon et al. 2011, 2015a), this dynamic between drifting and attached states may enhance stochasticity in the availability of propagules and thus the potential distribution of recruits.

Because recruits were concentrated on block edges, space available for new settlers could have been limited. However, the number of recruits did not decrease between the early summer sampling periods, suggesting that the available space on blocks for *Codium* recruitment was not limited. The apparent decrease in the number of recruits over the summer season may have resulted from growth of the recruits observed in the summer to juvenile and/or adult stages, coupled with limited recruitment during the summer months. Density-dependent effects (e.g. Arenas et al. 2002) could have also contributed to this decrease and include a higher vulnerability to grazers (Vadas et al. 1992), or a higher mortality of young stages of *Codium* due to a preferential grazing season (Underwood 1981). Gamete production elsewhere is known to occur at the beginning of the fall (Fralick and Mathieson 1973; Hanisak 1979) and could explain the observed temporal variation in recruitment, although observations made in the present study do not allow us to differentiate whether button stages originated from parthenogenesis (gametes) or utricles.

### Factors linked to *Codium* establishment

Rapid colonization of disturbed or non-vegetated areas and competition with native species are well-known mechanisms associated with the establishment of invasive macrophytes (Bando 2006; Scheibling and Gagnon 2006; Valentine et al. 2007; Williams 2007; Davidson et al. 2015). In this study, higher eelgrass density was associated with higher *Codium* abundance (recruits, juveniles and adults). Since there are little hard substrata in the study area, *Codium*’s association with eelgrass rhizomes is essential to its establishment. However, because this study used a standard recruitment substratum, it confirms that factors other than rhizome exposure can link *Codium* invasion to eelgrass density. Retention of seaweed fragments (Tweedley et al. 2008; Gagnon et al. 2015a) and smaller particles (Gacia et al. 1999) increases with eelgrass bed density due to flow reduction by the shoots. The close proximity of recruitment blocks to a nearby eelgrass canopy could have also allowed the eelgrass to sweep the surface of the blocks (Vadas et al. 1992), which could have a positive effect for algae in soft-bottom habitats as it could remove sediments and dislodge grazers (Choi 2003).

Some observations indicated that *Codium* had good survivorship once it settled and reached the recruit size. A demographic wave was observed on the blocks, as indicated by the consistently positive relationship between the number of recruits at one sampling date and that of subsequent size classes (i.e. recruits to juveniles; juveniles to adults) at subsequent sampling dates. Further, the number of fronds growing on a block at the end of the experiment was well predicted by the total cumulative number of recruits observed on the same block. A similar result was observed in a concomitant study conducted at a smaller scale (similar experimental layout but blocks spaced at 6 m) where recruits were observed on all the blocks, and only three of the 58 recruitment blocks showed a lower number of fronds than recruits at the end of the experiment (Drouin 2013). The link between the abundance of recruits and the subsequent stages may be a temporal autocorrelation artefact. However, the succession among life stages observed on blocks also suggests that factors regulating population demography, such as grazing and environmental conditions, likely did not have negative effects at these locations. The largest potential grazer in the study site was the common periwinkle, *Littorina littorea*, which has been shown to be able to limit recruitment of *Codium* by grazing small (<2 cm) and damaged fronds (Scheibling et al. 2008). However, this species was rarely observed on the blocks. The most abundant grazers associated with the blocks were snails of the genus *Calliostoma*, which are much smaller than *L. littorea*, but can reach a higher density on mature *Codium* thalli (up to 0.57 ind cm^−2^, Drouin et al. 2011). This snail, as for *L. littorea*, may be an efficient grazer on newly settled *Codium* but is likely ineffective on juveniles, i.e. >2 cm (Lubchenco and Gaines 1981; Scheibling et al. 2008). A decrease in the number of blocks with *Codium* between September 2008 and June 2009 clearly shows that some factors may not only limit *Codium* expansion within eelgrass beds but may also cause small scale retractions. One likely driver in this system is the burial of blocks by sediments, as was observed on blocks in deeper stations (>2 m), where the flow index was lower and the accumulation of sediments on block upper surfaces—where buttons usually appear—was observed (Drouin, pers. obs.). A low resistance to sedimentation could explain the observed loss of *Codium* on these blocks (Thomsen and McGlathery 2006, 2007). According to Thomsen et al. (2007), factors related to light extinction and sedimentation are likely strong limiting factors for *Codium* in soft-bottom habitats and can affect not only recruits but also adult stages. Due to the small range of light variation that we observed, the role of light could not be addressed by our data.

Biomass–density relationships may also partly explain the observed variation in *Codium* density on blocks. The densities of juveniles and adults were well predicted by the estimated biomass of *Codium* on blocks at the previous sampling date. The good correlation between biomass and density may indicate that the even-sized stands growing on blocks did not reach critical density levels, beyond which self-thinning would occur (Scrosati 2005). The capacity to form mature fronds while growing in crowded stands is likely a life history trait that promotes *Codium* establishment where suitable substrata and favourable growing conditions are present.

## Conclusion

By modifying habitat characteristics, species can promote the establishment of other species, both native and non-native (e.g. Altieri et al. 2010), and thus, invasion may be promoted through facilitation (Bulleri et al. 2008). In the present study, the facilitating eelgrass trait, i.e. its density, was not uniformly distributed, affecting the distribution of *Codium* within the invaded habitat. Moreover, in some areas, environmental conditions seemed to affect *Codium* survival, creating a mosaic of more or less adequate habitats for its establishment, although these factors still warrant further research. This study brings empirical evidence that habitat heterogeneity can promote invasion success as well as coexistence (Melbourne et al. 2007; Tamburello et al. 2013). Considering that the effects of invaders in general, and of *Codium* specifically (Drouin et al. 2012), are often dependent on their abundance and distribution, knowledge of where they can successfully establish within a given habitat is critical for understanding both their local and widespread impacts on invaded ecosystems.
